# GHz—THz Dielectric Properties of Flexible Matrix-Embedded BTO Nanoparticles

**DOI:** 10.3390/ma16031292

**Published:** 2023-02-02

**Authors:** Laura Mihai, Gabriel Caruntu, Aurelian Rotaru, Daniela Caruntu, Vasyl Mykhailovych, Cristina Elena Ciomaga, Nadejda Horchidan, Andrei Stancalie, Aurelian Marcu

**Affiliations:** 1 Center for Advanced Laser Technology, National Institute for Laser Plasma and Radiation Physics, 409 Atomistilor St., 077125 Magurele, Romania; 2 Department of Chemistry and Biochemistry, Central Michigan University, 1200 S. Franklin St., Mount Pleasant, MI 48859, USA; 3 Department of Electrical Engineering and Computer Science and MANSID Research Center, “Stefan Cel Mare” University, 13 Universitatii St., 720229 Suceava, Romania; 4 Department of Exact & Natural Sciences, Institute of Interdisciplinary Research, Al. I. Cuza University of Iasi, 11 Bld. Carol I, 700506 Iasi, Romania; 5 Faculty of Physics, Al. I. Cuza University of Iasi, 11 Bld. Carol I, 700506 Iasi, Romania

**Keywords:** BaTiO_3_, dielectric properties, THz spectroscopy, flexible dielectric materials

## Abstract

BaTiO_3_ (BTO) nanoparticles produced by wet chemistry methods were embedded in several types of flexible materials in order to fabricate flexible electronic devices. Starting from the produced nanoparticle dielectric properties, flexible material dielectric properties were tested for high electromagnetic frequencies (30 GHz–2 THz) using time domain spectroscopy. Dielectric performances of the different materials obtained with variable nanoparticle concentrations up to 40 wt.%, embedded in, gelatin, epoxy, and styrene-butadiene were compared at several working temperatures between 0 °C and 120 °C. Beside the general trend of ε′ decrease with temperature and loses increase with the operating frequency, we were able to identify few matrix dependent optimal nanoparticle concentrations. The best composite performances were achieved by the BTO-SBS matrix, with filler concentration of 2 wt.%, where the losses have been of 1.5%, followed by BTO-gelatin matrix, with filler concentration of 40 wt.%, with higher losses percent of almost 10% for THz frequencies.

## 1. Introduction

Ferroelectric materials are known to have high permittivity, spontaneously polarized domains, being able to switch in the presence of external fields [1]. Due to these properties, they are used or proposed for many applications in storage devices [2,3], sensors [4], memories, and many others. Within these materials, BaTiO_3_ (BTO) is the most intensively used as a ferroelectric material, having a low Curie temperature (120 °C). Moreover, it has been shown that it has tuning capabilities when used as dopant [5]. While it was initially considered as a “Pb free” piezoelectric alternative material, it also proved to be a convenient option for monitoring the structural phase transitions [6,7,8]. While polarization and surface charges are considered responsible for the material ferroelectric properties [8] (in direct correlation with material crystalline structure), the structural phase transitions are critical features controlling the dielectric parameters. 

From the applicative point of view, in the last years the demand for low power electronics and in particular for embedded flexible capacitors and resonators has increased. The requirements for these kind of devices are: low dimensions, flexibility, temperature stability and very good quality factor [9]. In spite of BTO promising dielectric properties, their permittivity is temperature dependent [9] and their mechanical properties (e.g., brittleness and thermal shock sensitivity and so on) could prevent it of being used for flexible devices in favor of polymers and other recently extensively studied materials with much more attractive mechanical properties. However, the dielectric properties of such materials are less competitive than BTO crystal properties. A good option proposed was to combine the two materials, by embedding BTO particles in such polymers. 

Different studies already presented in the literature report on the significant improvements of composite dielectric properties through this approach, respectively dielectric constant (ε′) values of 39 for PVDF-HFP-based materials [10] and ε′ of 15 for a polystyrene-based materials, but based on very high BTO nanoparticle concentrations, of 70% and respectively 40%. 

This work is focused on dielectric properties investigation and optimization, for different BTO nanoparticle-embedded flexible matrix. BTO nanoparticles were included in three different matrixes of gelatin, epoxy, and styrene-butadiene (SBS). We present here the fabrication methods as well as dielectric performances, both separate and in mixtures. While present technological demands, as well as telecommunication development trends, require higher and higher working frequencies. Presented results were obtained by THz–TDS spectroscopy technique, which proved to be a very useful tool for the study of dielectric properties and phase change within GHz–THz domains [11]. Dielectric parameters for the three polymer mixtures in different concentrations and at different working temperatures obtained by THz-TDS technique are further presented in the “Results” sections and a comparison of the three matrix results is included in the “Summary and Conclusion” Section.

## 2. Materials and Methods

### 2.1. Synthesis and Fabrication of Different Flexible Polymer-Ceramic Nanocomposites

BTO nanoparticles were initially fabricated using wet chemistry techniques. After a detailed nanoparticle characterization, from morphological, structural, and dielectric properties point of view, three flexible materials were chosen for embedding BTO nanoparticles: gelatin, epoxy resin and styrene-butadiene (SBS) polymers, in order to improve the polymeric matrix dielectric capabilities. Below, a brief description of each step is presented.

#### 2.1.1. BaTiO_3_ Nanoparticle Fabrication

Barium titanate nanoparticles were synthesized using the method previously reported in reference [12]. Shortly, 1 mmol of Ba(NO_3_)_2_ and 12.5 mmol of NaOH solution were mixed with a 1-BuOH solution containing 2.5 mL of oleic acid and 1 mmol of titanium butoxide (Ti(OBu)_4_) in butanol under vigorous stirring until a white creamy solution was formed. Subsequently 1-decanol was added to the final solution and stirred for 5 min. The obtained homogenic mixture was transferred into Teflon-liner and then placed into a stainless-steel autoclave. Then the autoclave with a reaction mixture was heated to 180 °C and maintained at this temperature for 18 h. After the end of the reaction the heating was stopped and the autoclave was cooled to ambient temperature naturally. Obtained nanoparticles were washed with ethanol and collected by centrifugation.

#### 2.1.2. BaTiO_3_-Gelatin Nanocomposites Preparation

BaTiO_3_-gelatin nanocomposite thick films investigated within this work have been prepared by a solution method as described in detail in Ref. [13]. Briefly, the BaTiO_3_ cubic nanoparticles, weighted in different amounts, were mixed in acetone by using a sonification bath. Solid gelatin powder from porcine skin (gel strength ~300 g Bloom, type A, Sigma Aldrich, St. Louis, MO, USA) was dissolved in deionized water by heating in a water-steam bath and then mixed with the BTO suspension under magnetic stirring at 60 °C/20 min. The slurry mixture was cast on Petri glass dishes and then dried for 72 h in a vacuum chamber at 25 °C to allow the complete solvent evaporation. In this way, a series of xBTO-(1-x)gelatin flexible nanocomposites with thickness of 200–350 μm and x = 0, 6.25 wt.%, 18 wt.%, 25 wt.% and 40 wt.% (e.g., with volume filling factors f = 0, 1.3 vol%, 4 vol%, 7 vol% and 12 vol%, respectively) have been produced.

#### 2.1.3. Epoxy Nanocomposite Preparation

The thermosetting EPR Epofix polymer used in the studies was cured at room temperature using Epofix hardener made by Struers—Ensuring Certainty (Denmark). EPR had a density of 1.1 g/cm^3^. Ferroelectric reinforcement BTO powders made in the solid state were employed as the active filler material. Composite thick films with different filler contents were prepared using a gravitational casting method, in order to result in flat composites with compositional gradient along the thickness direction and in-plane homogeneous composition. The samples have been prepared in the following way: the BTO powder in various compositions of 0 wt.%, 2.8 wt.%, 5.4 wt.%, 10 wt.% was mixed with acetone and ultrasonic bath for 30 min. The hardener solution was added and mixed for more than 15 min. Finally, the EPR solution was AQt mixed for another 5 min, in order to achieve homogeneous particle distribution within the polymer matrix, and then casted on the electrode substrates of Cu or Al foils to dry for 24 h in air.

#### 2.1.4. SBS Nanocomposite

In this case, the BaTiO_3_ nanoparticles were dispersed in toluene to form, highly stable and clear colloidal solutions. Separately, the solution of styrene-butadiene (SBS) was obtained by dissolving 10 mg of SBS granules in 10 mL of toluene followed by stirring on the plate at 40 °C until the clear solution was formed. Following, the BaTiO_3_/SBS composite was fabricated by mixing appropriate amount of BaTiO_3_ and SBS toluene solutions followed by casting on glass substrates. The SBS polymeric matrix was than generated with concentrations of the filler nanoparticles of 2, 5, and 10 wt.%. respectively. More details on composite fabrication are provided in Ref. [12].

### 2.2. Characterization Method

Dielectric properties of each sample are mentioned above and their dependencies on temperature were determined using time domain spectroscopy in THz range (THz-TDS). This method consists in measuring a reference waveform *E_r_*(t) with the THz beam passing through a sample diaphragm without any sample and a signal waveform *E_s_*(t) with the sample fixed and centered on the sample diaphragm.

The measured electric field corresponding to THz beam passing the sample, *E_s_*, in transmittance configuration, is determined using the following expression:

(1) $$E_{s}=E_{r}\frac{A_{s0}e^{j\varphi_{so}}}{A_{r0}e^{j\varphi_{ro}}}=T\left(n\right)e^{\frac{-\alpha d}{2}+\frac{ln\left(wd\right)}{c0}}$$

where *A_s_*_0_ and *A_r_*_0_ are the signal amplitudes for the sample and for the reference signal, *φ_s_*_0_ and *φ_r_*_0_ are THz pulse phases with and without the sample in the optical path, *α* is the absorption coefficient, *d* is sample thickness, *w* is the angular frequency, *c*_0_ is the speed of light, and *T(n)* is the losses from Fresnel reflectance on sample surface. 

The refractive index *n* depends on the THz wave phases when the reference and sample signals are acquired, and may be calculated by using the equation:

(2) $$n=1+\frac{c}{2\pi w}\left(\varphi_{s}-\varphi_{r}\right)$$

The material (generalized) permittivity *η*^ is defined as:

(3) η^(w)=ε^(w)+(iσ^(w)/ε0w)

where ε^=n^2, is the complex permittivity, with the complex refractive index *n*^ = *n* + ik and k = λα/4π = cα/2*w*. Thus, the relation between the dielectric constant, real ε′ and imaginary ε″ components, the refractive index *n* and absorption coefficient are given by:

(4) $$\varepsilon^{\prime }=n^{2}-k^{2}$$

(5) $$\varepsilon^{\prime }=2nk$$

While the “absorbance” and “refractive index” are parameters specific to “wave” propagation of an incident electromagnetic wave, material dielectric constant and dielectric losses are properties reflecting associated electric field propagation through the sample material. The real component *ε*′ is associated with the ohmic losses and *ε*″ is associated with the field “stored” energy. Such a “complex” coefficient also affects the wave vector, through the differential change of the propagation speed of the incident wave over different propagation directions within the sample material. 

In this study, knowing the dielectric components from Equations (4) and (5), it was possible to determine the relative quantity of material energy loss (denoted as loss tangent *tanδ*) defined as the ratio between the dielectric constants, imaginary *ε*′ and the real component *ε*″:

(6) $$tan\delta =\frac{\varepsilon^{\prime \prime }}{\varepsilon^{\prime }}=\frac{Energy\;lost}{Energy\;stored}$$

This ratio it is called also the material dissipation factor, and its reciprocal is called material quality factor.

In these studies, the power absorption coefficients and the refractive index for BaTiO_3_ pellet (NP size 10 nm) were measured using the method described above, in the frequencies range of 0.03–2 THz, using an average of 1800 scans, spectral resolution of 1 cm^−1^, with the attenuated total reflectance module of a TPS3000 THz spectrometer from TeraView. 

The dielectric properties (real and imaginary components), the phase shift, and material loss factor (*tanδ*) corresponding to each previously mentioned nanocomposite, have been determined using the transmittance configuration of the same equipment and with the same settings parameters. Each sample was placed in a mechanical holder having temperature control (0–120 °C), having the THz beam in its central point. For each sample, a reference signal has been registered outside the THz beam path. For transmittance measurements, water vapors were diminished by purging dry nitrogen in the sample chamber. A start delay time of 300 s was applied for all samples, in order to have water vapor cleaning for each reference and sample.

## 3. Results

### 3.1. BTO Properties

**Figure 1:**
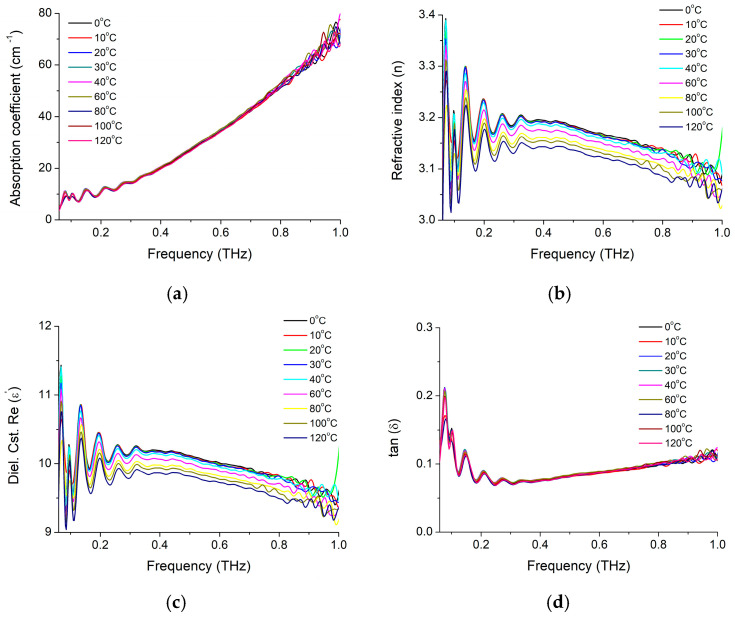
BaTiO_3_ nanoparticle frequency dependent (**a**) absorbance, (**b**) refractive index, (**c**) dielectric constant ε′, and (**d**) losses tangent (*tanδ*) at temperatures between 0 °C and 120 °C.

In order to evaluate dielectric properties in high frequency domain (GHz–THz), specific to the BaTiO_3_ fillers nanocrystals, before their insertion in different types of flexible nanocomposite, we first investigated pure BaTiO_3_ nanoparticle having 10 nm sizes by using time domain spectroscopy in frequencies range of 60 GHz–3 THz, as specified in the previous section. Measurements were performed at temperatures between 0 °C and 120 °C and the main results are presented in Figure 1. It can be noticed in Figure 1a that the values of absorption coefficient increase with frequency, with values up to 78 cm^−1^ corresponding to the higher frequencies. Similar to other literature results [14], no prominent absorption peaks have been observed in our case, but higher absorption coefficient values at the frequency of 1 THz. Global absorption features are not significantly changed by temperature variation in the selected range. However, the refractive index values, are decreasing around 20% with the temperature increase, but decrease with approximately 40% with the frequency (Figure 1b). 

For this sample, dielectric constant (ε′) is decreasing with about 10% in the investigated frequency range, being affected by temperature, with less than 10% variation within the 0–120 °C temperature range (Figure 1c). The tangent loss curve suggests an optimal working frequency around 350 GHz and 25% increase toward higher frequencies up to 1 THz and toward lower frequencies down to about 100 GHz. In other words, a three times higher or lower frequency will produce a similar increase of losses. Interesting to remark is the fact that within the optimal frequency range, a temperature increase does not seem to affect the losses with more than few percentages, while for frequencies outside of this range (particularly for lower frequencies), the losses (below 100 GHz) are changed up to 25%.

**Figure 2:**
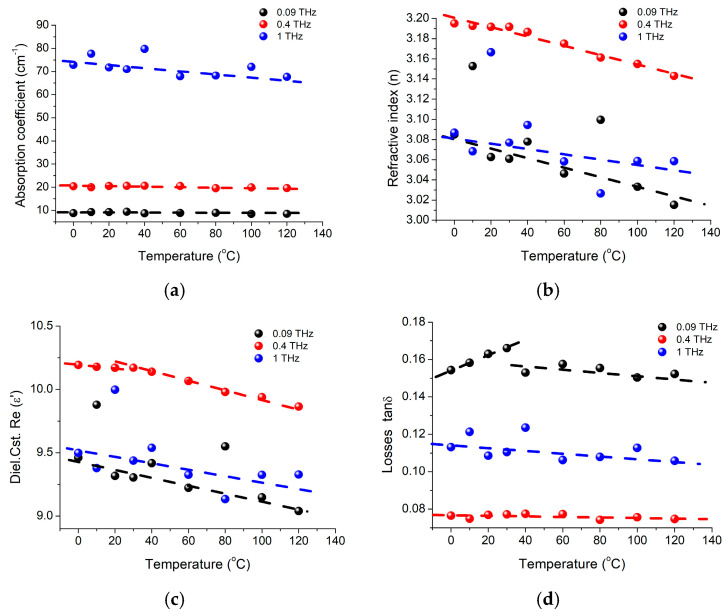
BaTiO_3_ nanoparticle temperature dependent response, at 3 frequencies of 90 GHz, 400 GHz and 1 THz, for (**a**) absorbance, (**b**) refractive index *n*, (**c**) dielectric constant ε′, and (**d**) losses *tanδ*.

To better understand the temperature impact on the BTO structure, we investigated the changes of its optical and electrical parameters in temperature range of 0 °C and 120 °C, for three specific frequencies: 90 GHz, 400 GHz, and 1 THz (Figure 2). It was noticed that even though the absorption coefficient does not seem to be significantly affected by the temperature increase at lower (GHz) frequencies, for frequencies above 1 THz, the absorption coefficient starts to gradually decrease for temperatures above 30 °C, as seen in Figure 1a. This trend is more visible in the case of refractive index, for the same temperature range, but for all selected frequency values (Figure 2b). While the dielectric constant variation curves (Figure 2c) are rather “noisy” and a clear “turning point” around 30 °C in the temperature dependence is obvious only for the 400 MHz variation curve, the losses variation for the three analyzed frequencies (Figure 2d) are showing the 30 °C temperature as a threshold temperature over which the losses will start increasing, particularly for frequencies further beyond 350 GHz.

**Figure 3:**
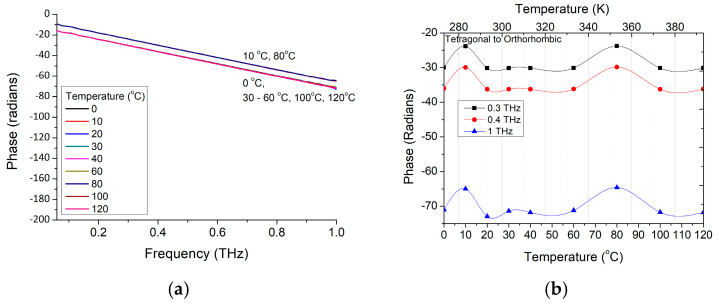
Phase changes in BaTiO_3_ nanoparticles (**a**) with frequency at different temperatures and (**b**) with temperature at three frequencies of 300 GHz, 400 GHz, and 1 THz.

Analyzing the ε′ dependence on temperature (Figure 2c), we can conclude that the decrease (e.g., from 10.19 to 9.86 at 400 GHz) is not related to the radiation absorption in material, but rather to refractive index (Figure 2b) and polarization changes, represented in Figure 3. It could be also observed that the phase is linearly decreasing with the frequency increase, but the curve is shifted at two temperatures, around 10 °C and 80 °C. The phase shift is generically associated with transition from ferroelectric tetragonal to ferroelectric orthorhombic phase present slightly above 0 °C [3], when an intrinsic polarization appears to change its orientation from parallel to the edge to parallel to the diagonal face of BaTiO_3_ crystal unit cell [15]. Consequently, this will imply a change in the refractive index and on losses tangent values, and of the dielectric properties on the propagation wave direction.

A phase change around 60–80 °C was also noticed, that seems to also have a contribution on ε′ values at lower frequencies; change that could be related to “superficial” (interface) tetragonal—cubic structure transition.

Starting from the pure BaTiO_3_ nanoparticles dielectric parameters, we tried to use it as a filler into different polymeric matrix for their applicability in flexible optoelectronic devices.

### 3.2. Dielectric Properties of Flexible Polymer-Ceramic Nanocomposites Based on BaTiO_3_ NP

#### 3.2.1. Gelatin Nanocomposite

**Figure 4:**
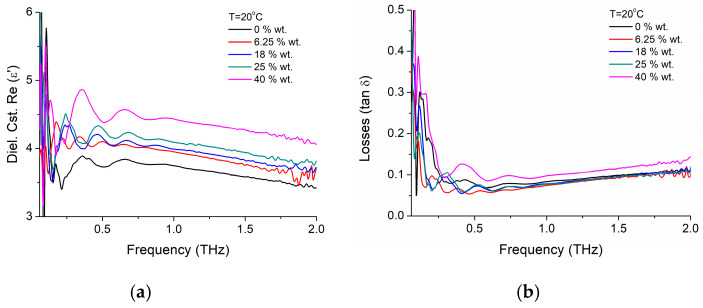
Room temperature frequency-dependent response of (**a**) dielectric constant e′ and (**b**) losses of xBTO-(1-x) gelatin composites thick films with 0 wt.%, 6.25 wt.%, 18 wt.%, 25 wt.%, and 40 wt.%.

In this work, we focused on a gelatin matrix xBTO-(1-x) Gel, with embedded BaTiO_3_ filler concentration of x = 0, 6.25, 18, 25, 40 wt.%. The gelatin nanocomposite dielectric properties in frequency range of 30 GHz–2 THz, and temperature variation between 0 °C and 120 °C are represented in Figure 4. In this case, the dielectric constant (Figure 4a) values for the real component ε′ decrease from values of 4–4.5 at 0.35 THz, to 3.8–4.3 values at 2 THz which represents about 40% of the pure BTO nanoparticle values, but a 10–20% increase from the pure gelatin performances for the same frequency range. Actually, the dielectric constant ε′ increases gradually with increasing the weight fraction of BTO in the gelatin matrix; for example, at 2 THz, we have ε′ values of 3.75 for x = 0 wt.%, 3.95 for x = 6.25 wt.%, 3.99 for x = 18 wt.%, 4.09 for x = 25 wt.%, and 4.44 for x = 40 wt.%. The observation is not surprising and is in good correlation with other literature results [14]. In terms of losses (Figure 4b), low values, around 0.1, were obtained for frequencies between 50 GHz and 500 GHz and about 1 for frequencies up to 2 GHz. The gelatin nanocomposite frequency dependence is relatively limited for this spectral domain and it also has an increasing tendency with the increase of nanoparticle concentration. Remarkably is the fact that, if an initial low concentration around 6% is decreasing the losses with more than 10% below the pure gelatin values, for some frequency intervals (e.g., frequencies below 500 GHz), an increase in nanoparticle concentration will continuously increase the losses, and, for higher NP concentration (e.g., 40%) could overcome with more than 10% the pure gelatin losses for the same frequency range.

**Figure 5:**
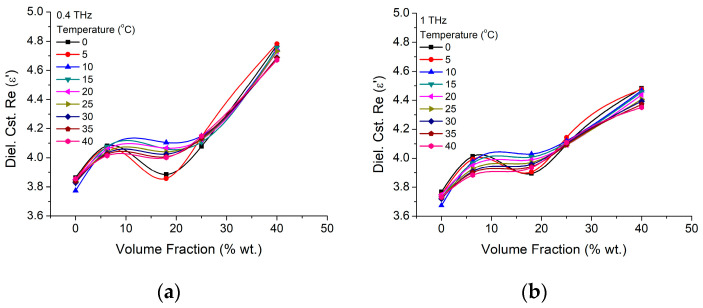
BTO-Gelatin dielectric constant ε′ variation with particle concentration volume fraction variation at (**a**) 400 GHz and (**b**) 1 THz and different temperatures.

**Figure 6:**
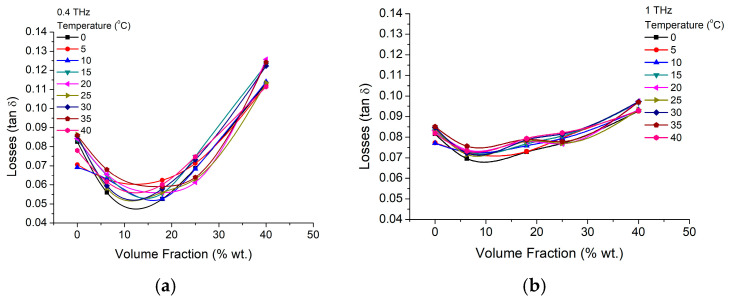
BTO-gelatin losses variation with particle concentration volume fraction variation at (**a**) 400 GHz and (**b**) 1 THz and different temperatures.

For a better understanding of the parameter dependence on the nanoparticle concentration, the dielectric constant and losses are presented as a function of volume fraction, for temperatures between 0 °C and 40 °C, in Figure 5 and Figure 6, for two frequencies of 400 GHz and 1 THz. Here, it could be easily observed, that low concentration of nanoparticles (~6%) have a more efficient impact on the dielectric constant, while the temperature increases over 10 °C decrease will further enhance that increase, further than the gelatin intrinsic values (0%). At higher concentrations, the nanoparticles will start to interact to each-other, diminishing the BTO-Gelatin interface area. As a consequence, the nanoparticle surface can charge the initial trend, matrix dielectric performances having a larger impact on the BTO crystal dielectric performances. We estimate that this process starts when the nanoparticle concentrations are higher, 10 wt.%. Higher frequencies (1 THz) seem to diminish the trend without actually modifying it. However, in terms of losses, (Figure 6) both, lower (400 GHz—Figure 6a) and higher frequencies (Figure 6b) suggest that the nanocomposite in lower concentrations of BTO is more efficient. Thus, if at lower frequencies, nanoparticle concentrations between 5% and 10% seems to be preferable, at higher frequencies, with the increase of the concentration above 6% the losses constantly increase, while the temperature increase just make the losses higher, depending on the operating frequency and particle concentration, with up to 10% at higher frequencies and 20% at lower frequencies, and just within 20–30 temperature degrees variations. The fact that the losses values are in the range of pure BTO nanoparticle or slightly lower is remarkable.

**Figure 7:**
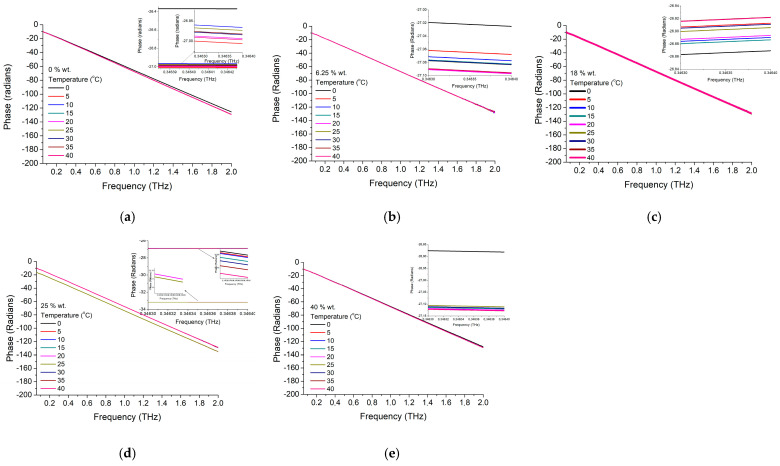
BTO-gelatin phase variation with frequency at (**a**) 0 wt.%, (**b**) 6.25 wt.%, (**c**) 18 wt.%, (**d**) 25 wt.%, and (**e**) 40 wt.%.

While looking at the phase shift, we noticed a similar trend of variation with frequency (a linear variation), a similar starting point for lower frequencies (~50 GHz) and a similar slope, respectively ~70 radians phase shift at 1 THz. Interesting to underline is the fact that, in this case, the curve shift is no longer visible only at 20 °C and 80 °C as in the pure nanoparticle case, but it depends on the nanoparticle concentration as can be seen in the insets from Figure 7.

**Figure 8:**
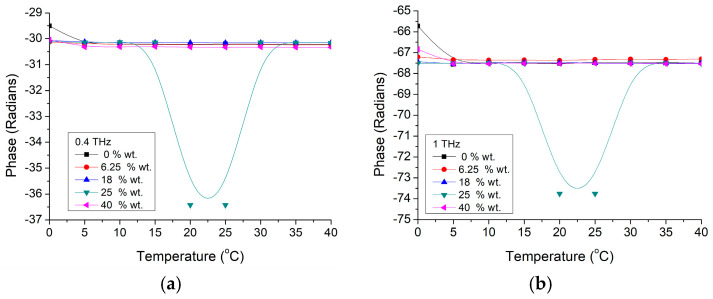
BTO-gelatin phase variation with temperature at two frequencies of (**a**) 400 GHz and (**b**) 1 THz.

A more detailed investigation has been performed by analyzing the phase shift with the temperature of BTO-Gelatin at two different frequencies of 400 GHz and 1 THz for different nanoparticle concentrations, as presented in Figure 8. It could be noticed that in both cases, the reference phase shift for the two frequencies is slightly decreased with just a few radians comparing with pure nanoparticle case. In this case, we have a phase shift decrease just below 5 °C (with shift amplitude depending both on nanoparticle concentration and working frequency) while the 5 radians positive shift around 20 °C becomes a large negative shift of more than 30 radians around the same temperature. This could indicate that the phase transformation is different more likely due to the interfaces between BTO nanoparticles and gelatin material, while gelatin itself does not have any intrinsic contribution on that shift around the mentioned temperature range, at any of the two presented frequencies of 400 GHz (Figure 8a) and 1 THz (Figure 8b).

#### 3.2.2. Epoxy Nanocomposite

**Figure 9:**
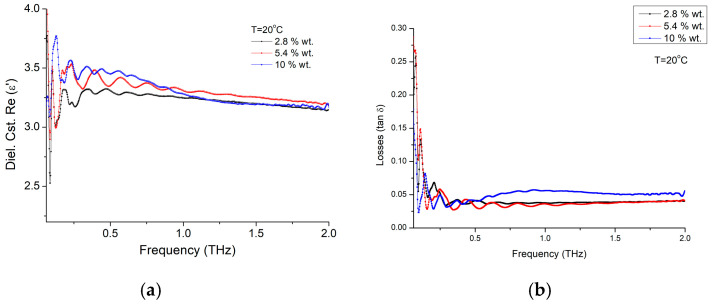
BTO-epoxy film composite (**a**) dielectric constant ε′ and (**b**) losses variation with frequency at room temperature, for different nanoparticle concentrations of 2.8 wt.%, 5.4 wt.% and 10 wt.%.

The following nanocomposite material studied was the epoxy. The dielectric constant, real part ε′, and material losses at room temperature variations with the frequency are represented in Figure 9a for three concentrations of BTO nanoparticles embedded in epoxy: 2.8 wt.%, 5.4 wt.%, and 10 wt.%. The general trend is rather similar to the pure nanoparticles, with a similarly increasing trend toward higher nanoparticle concentration (e.g., 10%), but with values around three times smaller within this composite material. On the other hand, material losses (Figure 9b) are about half of the pure nanoparticles’ values, while frequency dependence is rather week for frequencies above a few hundreds of GHz. However, at higher concentrations (e.g., 10 wt.%) the losses have an increasing trend between 300 GHz and 800 GHz, in a rather similar way as the pure nanoparticles. However, increasing trend similarity is not preserved at higher frequencies, and has a constant value after 1 THz.

**Figure 10:**
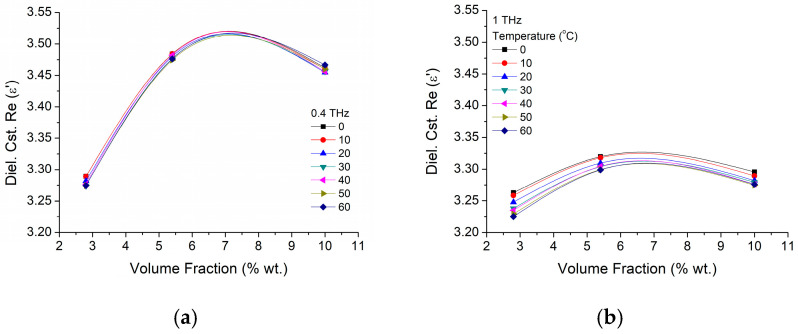
BTO-epoxy dielectric constant real part (ε′) variation on nanoparticle volume fraction variation at different frequencies of (**a**) 400 GHz and (**b**) 1 THz.

A clearer analysis of dielectric constant dependence on the particle concentration is presented in Figure 10 for two working frequencies of 400 GHz (Figure 10a) and 1 THz (Figure 10b). It could be observed that for both frequencies the dielectric constant has an increasing trend at low particle concentrations (~5 wt.%), while for higher concentration the dielectric values decrease. Having a look over the temperature influence we could notice that its increase will only diminish ε′ without changing its trend, and this happens more clearly at higher frequencies, where, in spite of the smaller coefficient peak values at higher versus smaller frequencies (~3.32 versus ~3.52), the decrease is about double (0.04–0.08 versus 0.02–0.03).

**Figure 11:**
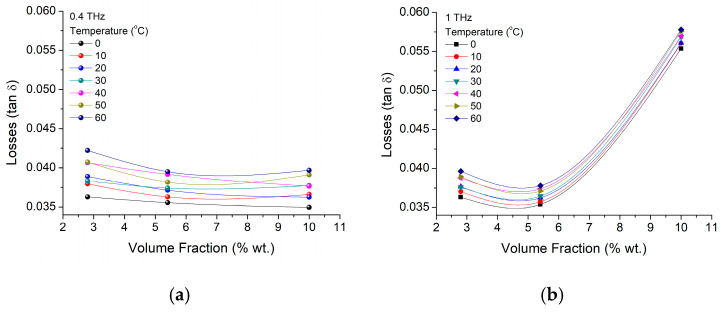
BTO-epoxy losses variation on nanoparticle volume fraction variation at frequencies of (**a**) 400 GHz and (**b**) 1 THz for different temperatures.

In terms of losses, a similar analysis performed as in the case of the dielectric constant for the 400 GHz and 1 THz, has shown that, while at lower frequencies (e.g., 400 GHz in Figure 11a) particle concentration does not have a critical influence, at higher frequencies (e.g., 1 THz—Figure 11b), smaller concentration has a slightly diminishing effect on the losses (below 10 wt.%) while at higher concentration it has a clear increasing effect on losses (up to 50 wt.% at 10 wt.%).

**Figure 12:**
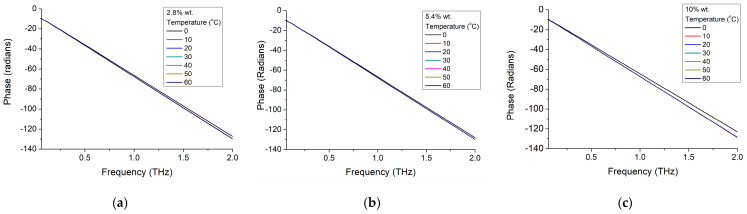
BTO-epoxy phase variation with frequency for different nanoparticle concentrations of (**a**) 2.8%, (**b**) 5.4%, and (**c**) 10% at different temperatures.

**Figure 13:**
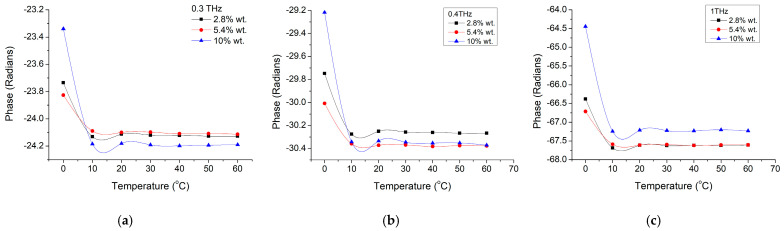
BTO-epoxy phase variation on temperature at different frequencies of (**a**) 300 GHz, (**b**) 400 GHz, and (**c**) 1 THz.

By analyzing the phase shift dependence on frequency (Figure 12), we could notice a similar trend of the composite material with the original BTO nanoparticles. It has a linear dependence, quasi-independent on the ambient temperature, with similar values with the phase shift of the pure nanoparticles. However, the slope is slightly dependent on the nanoparticle concentration, as observable while comparing Figure 12a–c for the three nanoparticle concentrations of 2.8 wt.%, 5.4 wt.%, and 10 wt.%. While comparing phase shifting only for 3 working frequencies of 300 GHz (Figure 13a), 400 GHz (Figure 13b) and (Figure 13c) it could be more clearly observed that only lower temperatures below 20 °C have some influence on the phase shift and particularly on higher concentration materials (10 wt.%). It should be remarked that while in the previous cases the change was around 20 °C–30 °C, this time the change is at lower temperatures, close to the bulk reported material phase transition (tetragonal—orthorhombic) temperature of 0 °C [3]. Furthermore, taking into account that the phase shift at 0 °C is also increasing with the nanoparticle concentration, this shift is smaller (below 1.5%) when compared with the pure nanoparticles shift (about 15%). Thus, a quasi-linear correlation of the phase shift with the nanoparticle concentration could be established for low operation temperatures (around 0 °C). 

**Figure 14:**
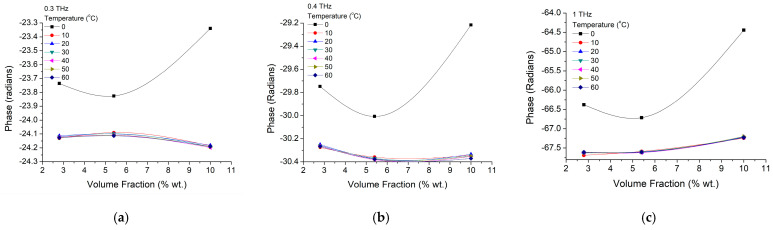
BTO-epoxy phase on nanoparticle volume fraction variation at different working frequencies of (**a**) 300 GHz, (**b**) 400 GHz, and (**c**) 1 THz and different temperatures.

Figure 14 presents the phase shift dependence on nanoparticle concentration for three working frequencies of 300 GHz, 400 GHz, and 1 THz—Figure 14c. It is observed that the phase shift has a weak dependence on concentration, but the trend is strongly dependent on the working frequencies. Thus, if the phase shift is negative with the particle concentration at lower frequencies of 300 GH–400 GHZ, for higher frequencies of 1 THz the shift became positive, while the absolute value remains within the few percent orders, for the investigated frequency range. It is also interesting that the temperature does not really influence the phase shift above 0 °C, suggesting that only in the orthorhombic phase, nanoparticle concentration and working frequencies play an active role.

#### 3.2.3. SBS Nanocomposite

**Figure 15:**
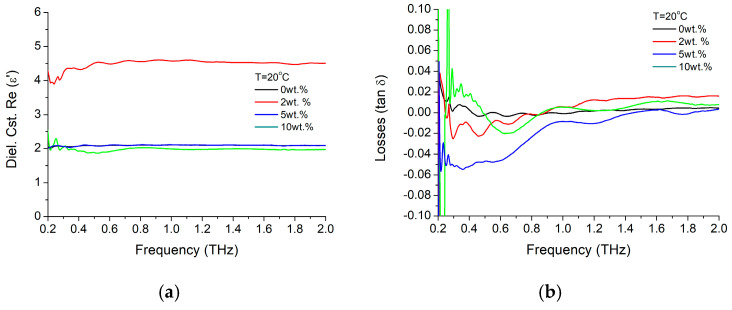
BTO-SBS film composite (**a**) dielectric constant ε′ and (**b**) losses variations with frequency at room temperature, for 2 wt.%, 5 wt.% and 10 wt.%.

The dielectric properties of BTO-SBS nanocomposite material with embedded BaTiO_3_ into a SBS polymeric matrix, with concentrations of the filler nanoparticles of 2, 5, and 10 wt.%. have been further investigated and the dielectric constant and material losses variation at room temperature with the working frequency are further presented in Figure 15. As it could be seen in Figure 15a, the dielectric constant has a value quasi-independent on the working frequency at least for values above 500 GHz, but dependent on the nanoparticle concentration. Thus, while big concentrations provide dielectric constant values (~2) about five times smaller than the pure nanoparticle’s one (~10), at small nanoparticle concentrations (2 wt.%), the dielectric constant ε′ is doubling its value (>4). However, it is interesting to mention that toward smaller frequencies (~200 GHz), volume fraction of 2 wt.% and 10 wt.% tends to have opposite trends but with relatively small variations up to 10%. In terms of material losses, composite material tends to have values smaller than the pure nanoparticle values. As trends, we should notice that for high NP concentration (10 wt.%), for some reason doubling of the dielectric constant ε′ is accomplished by a slight increase of losses. On the other hand, we should remark the tendency of negative losses values for lower frequencies and high concentration of nanoparticles, suggesting a rather “inductive” behavior of the composite material for higher NP concentrations.

**Figure 16:**
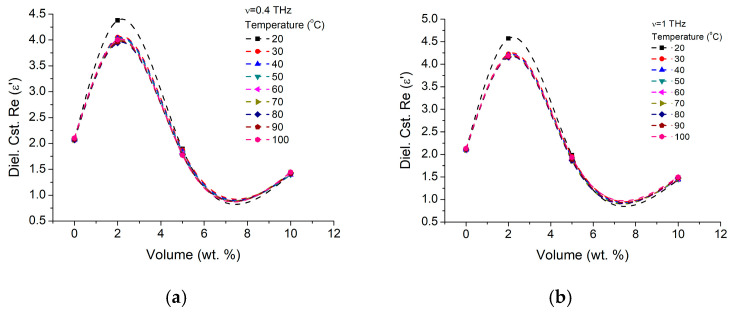
BTO-SBS dielectric constant real part (ε′) variation on nanoparticle volume fraction, at different temperatures for (**a**) 400 GHz and (**b**) 1 THz.

**Figure 17:**
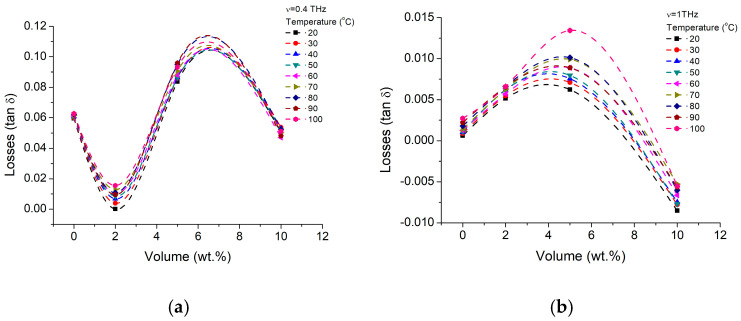
BTO-SBS losses variation with BTO nanoparticles volume fraction for two working frequencies of (**a**) 400 GHz and (**b**) 1 THz, at different working temperatures.

A dielectric constant dependence on NP concentration is presented in Figure 16 for different temperatures between 0 °C and 120 °C and two working frequencies of 400 GHz and 1 THz. It could be seen that the 2 wt.% concentration is inducing the dielectric constant increase not only at both frequencies (as previously pointed out in Figure 15a) but also for all temperature in the investigated range. Even when the BTO changes from tetragonal to orthorhombic phase, ε′ increase is preserved (0 °C curve). Further increase in the NP concentration will actually decrease the ε′ value below the pure SBS values (and 10 times below the pure NP nanoparticles). A similar analysis over the dielectric losses (Figure 16), shows that, at lower frequencies (400 MHz) the increase of the dielectric constant ε′ is accomplished by a decrease in dielectric losses for the 2 wt.% NP concentration (Figure 17a). At further concentration increase (5 wt.%), losses reach values over SBS original losses values, and at further increase (10 °C) losses return to the initial values. At higher operating frequencies (1 THz), dielectric losses increase with the NP concentrations (Figure 17b) up to concentrations of 5 wt.% and decrease at much higher NP concentrations (10 wt.%). While temperature increase is only going to increase the losses the understanding of such behavior might relay only on the role played by the NP-SBS interfaces. 

**Figure 18:**
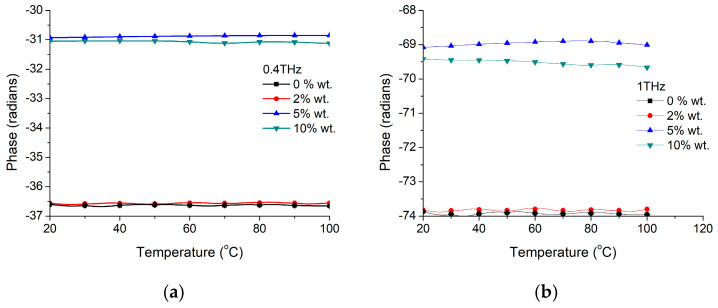
Phase change with temperature in BTO-SBS nanocomposite having different filler concentrations (0 wt.%, 2 wt.%, 5 wt.%, 10 wt.%) at different frequencies of: (**a**) 0.4 THz, (**b**) 1 THz.

**Figure 19:**
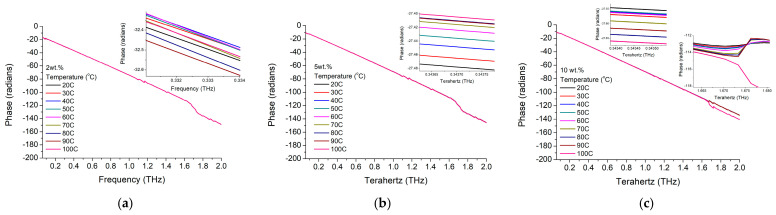
BTO-SBS phase variation with frequency for different BTO nanoparticle concentrations ((**a**) 2 wt.%, (**b**) 5 wt.%, and (**c**) 10 wt.%) and different temperatures.

**Figure 20:**
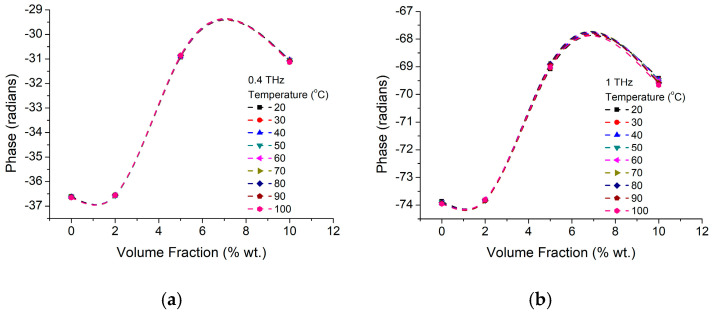
BTO-SBS phase variation with BTO nanoparticles volume fraction for two working frequencies of (**a**) 400 GHz and (**b**) 1 THz at different working temperature.

Phase change of the propagating wave is almost temperature independent both at lower (400 GHz—Figure 18a) and higher (1 THz—Figure 18b) working frequencies for all investigated BTO NP concentrations. However, composite phase shift dependence on the working frequency (Figure 19) shows a non-linear behavior around 1.675 THz (Figure 19c inset), unlike the pure NP or previously studied BTO NP composites. Furthermore, the return of this composite material to the linear behavior over the whole frequency range (up to 2 THz) could be achieved only for high NP concentration (of 10 wt.%) and 100 °C temperature, temperature closing tetragonal—cubic BTO phase transition. On the other hand, by comparing the behavior at two given frequencies of 400 GHz and 1 THz (Figure 20) we could notice that the wave propagation phase changes somewhere between 2 wt.% and 5 wt.% while further increase does not contribute, unless we have high temperatures (100 °C). Furthermore, some existing preliminary data (not presented here) suggest that this change is enhanced toward lower temperatures with increase in the BTO bulk contribution through the increase in the nanoparticle size. In other words, we attribute the change in the wave propagation phase and respectively material losses at higher frequencies and NP concentration to the changes in the NP structure toward cubic phase.

## 4. Summary and Conclusions

A summary of the dielectric constant (real and imaginary components) for all studied samples for the selected frequency range of 60–1 THz: pure BaTiO_3_, pure SBS membrane, pure gelatin membrane, BTO—gelatin nanocomposite with different weight percent of BTO filler (6.25%, 18%, 25% and 40 wt.%.) and BTO—epoxy with different weight percent of BTO filler (2.8%, 5.4% and 10 wt.%.) and BTO—SBS nanocomposite with different weight percent for BTO filler (2%, 5% and 10 wt.%.) is presented in Table 1. 

Evaluating the storage capabilities for all samples, we found that, gelatin has the highest dielectric values (3.43) but with the largest losses percent (10%), followed by epoxy with 2.8 and losses of around 5% from total storage. The best composite performances were achieved by the BTO-SBS matrix, with filler weight of 2%, where the losses have been of 1.5%, followed by BTO-gelatin matrix, with filler weight of 40%, but with higher losses percent of almost 10%.

Composite dielectric performances in comparison with the pure nanoparticles and each pure matrix performances are presented in the sections above. Changes in the dielectric parameters are in principle attributed to the NP-matrix interfaces, both in the sense of dielectric performances and in BTO phase-change (temperature) transition points, as commented in each section above. However, further studies on the surface versus volume ratio influences over the composite material dielectric performances are necessary for a better understanding and control of the material performances.

## Figures and Tables

**Table 1:** Dielectric properties of different matrix-embedded BTO nanoparticles.

Sample	ε′	ε″
BTO pellet 900 μm thickness		
100%	9–11.5 (0.06–1 THz), 9.45 (1 THz)	0.5–2 (0.06–1 THz), 0.99 (1 THz)
BTO—SBS		
0%	1–2.5 (0.06–2 THz), 2.1 (1 THz)	0.001–0.6 (0.06–2 THz), 0.007 (2 THz)
2% 10 nm	1.7–5.3 (0.06–2 THz), 4.1–4.5 (2 THz)	0.1–1.6 (0.06–2 THz), 0.07 (2 THz)
2% 20 nm	0.8–2.7 (0.06–2 THz), 2 (2 THz)	0.4–0.5 (0.06–2 THz), 0.025 (2 THz)
5% 10 nm	0.3–7 (0.06–0.1 THz), 1.96 (2 THz)	0.3–5.5 (0.06–0.1 THz), 0.3 (0.1–2 THz)
5% 20 nm	0.2–6.5 (0.06–0.1 THz), 1.95 (2 THz)	0.1–2.4 (0.06–2 THz), 0.016 (2 THz)
10% 10 nm	0.6–2.4 (0.06–2 THz) 1.44 (2 THz)	0–0.7 (0.06–2 THz) 0.007 (2 THz)
10% 20 nm	3.8–0.04 (0.06–2 THz) 1.35 (2 THz)	0.05–2 (0.06–2 THz) 0.002 (2 THz)
BTO—Gelatin		
0%	2.2–5.7 (0.06–2 THz), 3.43 (2 THz)	0.1–1.5 (0.06–2 THz), 0.37 (2 THz)
6.25%	3.5–4.5 (0.06–2 THz), 3.66 (2 THz)	0.2–1.6 (0.06–2 THz), 0.34 (2 THz)
18%	3.5–5 (0.06–2 THz), 3.55 (2 THz)	0.2–1 (0.06–2 THz), 0.37 (2 THz)
25%	3.6–5.3 (0.06–2 THz), 3.9 (2 THz)	0.3–1.3 (0.06–2 THz), 0.44 (2 THz)
40%	3.9–6.6 (0.06–2 THz), 4.11 (2 THz)	0.4–2.4 (0.06–2 THz), 0.53 (2 THz)
BTO- epoxy		
0%	2.6–2.8 (0.06–2 THz) [16]	0–0.15 (0.06–2 THz) [16]
2.8%	2.6–3.5 (0.06–2 THz), 3.16 (2 THz)	0.1–0.4 (0.06–2 THz), 0.13 (2 THz)
5.4%	2.9–4.2 (0.06–2 THz), 3.2 (2 THz)	0.09–1.4 (0.06–2 THz), 0.13 (2 THz)
10%	3–3.7 (0.06–2 THz), 3.19 (2 THz)	0.08–0.7 (0.06–2 THz), 0.16 (2 THz)

## Data Availability

Not applicable.
